# Comparison of kV Orthogonal Radiographs and kV-Cone-Beam Computed Tomography Image-Guided Radiotherapy Methods With and Without Implanted Fiducials in Prostate Cancer

**DOI:** 10.7759/cureus.9916

**Published:** 2020-08-21

**Authors:** Mehmet F Eren, Didem Çolpan Öksüz, Mutlay Sayan, Songül Karaçam, Irina Vergalasova, Ayfer Ay Eren, Fazilet Öner Dinçbaş

**Affiliations:** 1 Radiation Oncology, Marmara University Pendik Education and Research Hospital, Istanbul, TUR; 2 Radiation Oncology, Cerrahpasa Medical Faculty, Istanbul University-Cerrahpaşa, Istanbul, TUR; 3 Radiation Oncology, Rutgers Cancer Institute of New Jersey, New Brunswick, USA; 4 Radiation Oncology, Saglik Bilimleri University, Kartal Dr. Lutfi Kirdar Education and Research Hospital, Istanbul, TUR

**Keywords:** prostate cancer, fiducial, imrt, igrt, kv-cbct

## Abstract

Introduction

The aim of this study is to investigate the performance of kilovoltage (kV) cone-beam computed tomography (CBCT)-based adjustments with respect to kV-orthogonal fiducial marker-based matching in a group of patients with prostate cancer.

Methods

Twenty prostate cancer patients were evaluated retrospectively: 10 with implanted fiducial markers and 10 without. Daily orthogonal kV imaging was recorded prior to radiation delivery. Images were evaluated in the left-right (LR), anterior-posterior (AP), and superior-inferior (SI) directions by matching either the implanted fiducials or going off bony anatomy, depending on the presence or absence of markers, respectively. Cone-beam computed tomography (CBCT) imaging was also subsequently acquired and images were aligned with the planning CT. The couch shifts were calculated and the patient’s position was adjusted accordingly. Standard deviations and random errors were also computed. Pearson correlation and Bland-Altman analysis were performed to evaluate relationships between the datasets.

Results

A total of 240 images were evaluated. The Pearson correlation coefficient for shifts applied to patients with markers using kV and CBCT was 88.3%, 87.8%, and 94.5% for the LR, AP, and SI directions, respectively. For those without markers, the respective values for the LR, AP, and SI directions were: 39.3%, 22.4%, and 3.7%. A Bland-Altman analysis comparing kV and CBCT in patients with markers, revealed R^2^ values of 0.152, 0.282, and 0.097 in the LR, AP, and SI directions, respectively. The R^2^ values for patients without markers were 0.008, 0.01, and 0.057, in the LR, AP, and SI directions, respectively.

Conclusions

Our data suggest that CBCT can be a viable option for image-guidance in clinical settings where fiducial markers are unavailable such as situations of inaccessibility or medical contraindications.

## Introduction

Early-stage prostate cancer can be effectively managed via 3D conformal radiotherapy (3DCRT) or intensity-modulated radiation therapy (IMRT). Radiation dose escalation studies have shown an improved biochemical relapse-free survival with higher doses compared to standard dose regimens [1-3]. However, dose escalation may result in adverse effects, with rectal and bladder toxicities being the two major limiting factors [4-5]. In order to maximize the therapeutic ratio, adjacent normal tissues should be spared as much as possible. However, a major challenge in the accurate targeting of the prostate gland is that it is very susceptible to motion in every direction, mainly due to the varying degrees of the fullness of neighboring structures, i.e. the bladder and rectum. Thus, in order to deliver an optimal treatment plan to the patient, it is necessary to incorporate corrective techniques in order to evaluate the inevitable organ mobility that will occur during the course of treatment [6], such as megavoltage (MV) imaging, kV imaging, ultrasonography (USG), kV or MV cone-beam computed tomography (CBCT) imaging, kV fluoroscopy, radiofrequency, and/or optical methods. Bony anatomy matching is a common procedure used to align prostate patients before treatment delivery, but its accuracy is limited by the gland’s mobility relative to the bones [6]. To overcome this problem, implanted gold markers have been used to detect the exact position of the prostate on radiographs when using MV or kV portal imaging systems. However, a limitation of using these markers is the required invasiveness of the implantation process, as well as the additional cost burden to the patient and social security system. Following the recent advent of CT technology mounted onto treatment linacs, CBCT has been utilized for image-guided radiotherapy (IGRT), which has resulted in improved accuracy for three-dimensional target volume and soft tissue visualization. However, the relative performance of CBCT in comparison to fiducial marker radiograph localization has not yet been established. Herein, we aim to compare kV CBCT-based prostate alignment with respect to kV radiograph-based fiducial marker alignment.

## Materials and methods

Twenty patients with low- to intermediate-risk prostate adenocarcinoma were treated with IMRT at Cerrahpaşa Medical Faculty, Istanbul. The study has been approved by the Institutional Ethical Committee (No: 22.10.2010 / 31782). Patient characteristics are presented in Table 1. All patients gave written informed consent to participate in the study. Treatments were delivered to the planning target volume (PTV). The PTV was defined with a 5 mm margin posteriorly and 8 mm in all other dimensions from the clinical target volume (CTV). The protocol requires that at least 95% (D95) of the PTV receives 76 Gy of radiation with a daily fraction size of 2 Gy.

**Table 1:** Patient characteristics PSA: prostate-specific antigen

		N
Gleason score	<6	5
	7	14
	>8	1
PSA	<10	4
	10-20	13
	>20	3
Total number of patients	20	
Median age	57	

Simulation

Patients were asked to have a half-full bladder and an empty rectum during the simulation and for the duration of radiotherapy. In order to localize the prostate, three gold fiducial markers (24k, 3 × 0.8 mm, CIVCO Medical Solutions, Kalona, Iowa) were implanted into the prostate via transrectal ultrasound (TRUS) guidance for 10 patients before treatment simulation and planning. Simulations were acquired in the supine position, with a leg immobilization device (Combifix-Sinmed, Civco, Kalona, IA). CT scans with contrast were acquired of the pelvis, with axial slices of 2.5 mm in thickness. The clinical target volume (CTV), planning target volume (PTV), fiducial markers, bladder, and rectum were contoured using the Eclipse treatment planning system (Varian Medical Systems, Palo Alto, CA).

Treatments

An IMRT technique was employed using 6 MV photons. All patients were treated using the Clinac DHX linear accelerator (Varian Medical Systems). Prior to daily treatment delivery, orthogonal images were captured using a kV imaging system (On-Board Imager® - OBI, Varian Medical Systems). During treatment planning, implanted fiducial markers were contoured on the CT and then projected onto the anterior and lateral DRR images, which were then used for registration with the acquired on-board kV images via manipulation in the superior-inferior (SI), anterior-posterior (AP) and left-right (LR) directions The deviation of the isocenter was calculated and recorded in LR, AP, and SI coordinates. Afterward, kV-CBCT was acquired with a slice thickness of 2.5 mm with the pelvis spotlight mode protocol, 512 by 512 FOV, and full-bowtie fan mount. The isocenter shifts were manually determined at the treatment machine before each treatment by the treating physician, by aligning soft-tissue structures between the CBCT and simulation CT. The deviation of the isocenter was measured in the LR, SI, and AP dimensions. Shifts from the CBCT were recorded for analysis but were never applied to patient treatment. kV imaging was repeated for all patients, including those with or without markers, using the above-mentioned method for consecutive five days (Table 2). Manual adjustments to the couch were performed if the recommended correction was > 3 mm in any of the cardinal directions. If a shift was executed, a second set of images was acquired for verification.

**Table 2:** Workflow description Workflow description of the imaging acquired and shifts applied between the two study groups, with and without implanted fiducial markers

Group		With Marker	Without Marker
Procedure			
kV imaging		+	+
	Alignment	With marker matching	With reference to bone anatomy
	Frequency	Daily	Daily
CBCT		+	+
	Alignment	With reference to soft tissue	With reference to soft tissue
	Frequency	Daily for the first 5 days, followed by weekly therapies	Daily throughout the treatment period
	Shift	Manual correction by the physician if deviation>3 mm	Automatic correction by the treatment table if deviation <3 mm

Statistical analysis

Data were analyzed using Statistical Package for the Social Sciences (SPSS) software, version 15.0 (SPSS Inc., Chicago, IL). The two IGRT methods were compared statistically using Pearson correlation and Bland-Altman analyses. A correlation analysis was performed to determine the level (extend-intensity rating) and direction of the relationship between the two variables. The correlation between variables was determined using the Pearson correlation coefficient where both variables were deemed continuous and from a normal distribution. This test investigated the correlation of couch shifts between CBCT and orthogonal kV radiographs. The calculated correlation coefficient was explained as the linear relationship between the aforementioned variables. Standard deviation values were calculated for all patients with and without markers in all three shift dimensions (LR, AP, and SI). Standard deviation values were interpreted as systematic errors. Random error values were calculated by taking the square root of standard deviation values. Mean and standard deviation values of absolute differences between the two imaging techniques were also compared.

## Results

**Figure 1:**
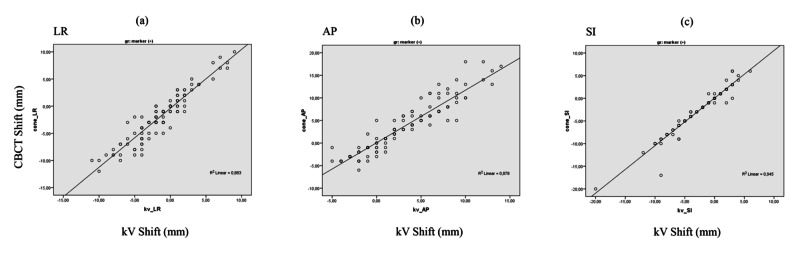
Pearson correlations for 10 patients with markers Pearson correlations of the applied kilovoltage (kV) shifts vs cone-beam computed tomography (CBCT) shifts (a-c) for 10 patients with markers in the left/right (LR), anterior/posterior (AP), and superior/inferior (SI) directions

**Figure 2:**
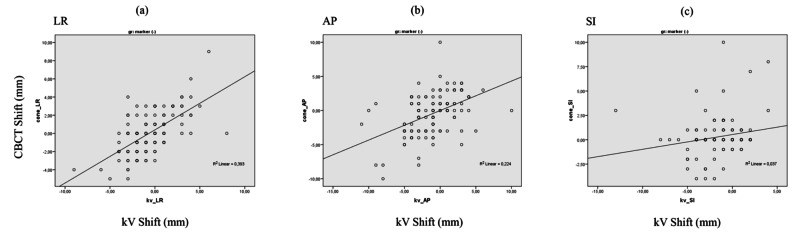
Pearson correlations for 10 patients without markers Pearson correlations of the applied kilovoltage (kV) shifts vs cone-beam computed tomography (CBCT) shifts (a-c) for 10 patients without markers and all fractions in the LR, AP, and SI directions

This study analyzed data from 20 patients. A total of 240 kV radiograph and cone-beam CT images were evaluated. A linear regression analysis found that Pearson’s correlation coefficient values (R2) between shifts for 10 patients with markers were 0.883, 0.878, and 0.945 for LR, AP, and SI, respectively (Figure 1a-1c). Also, the Pearson correlation coefficient values (R2) between shifts for the remaining 10 patients without markers were 0.393, 0.224, and 0.037 for LR, AP, and SI, respectively (Figures 2a-2c). The Pearson coefficient of determination “R2” indicated the degree of consistency between kV and CBCT for patients with/without markers, correlatively. It is always between 0 and 1. A higher value is better. As a result, it is evident that this data demonstrates a strong correlation between kV and CBCT for patients with implanted markers according to the Pearson correlation analysis. 

**Figure 3:**
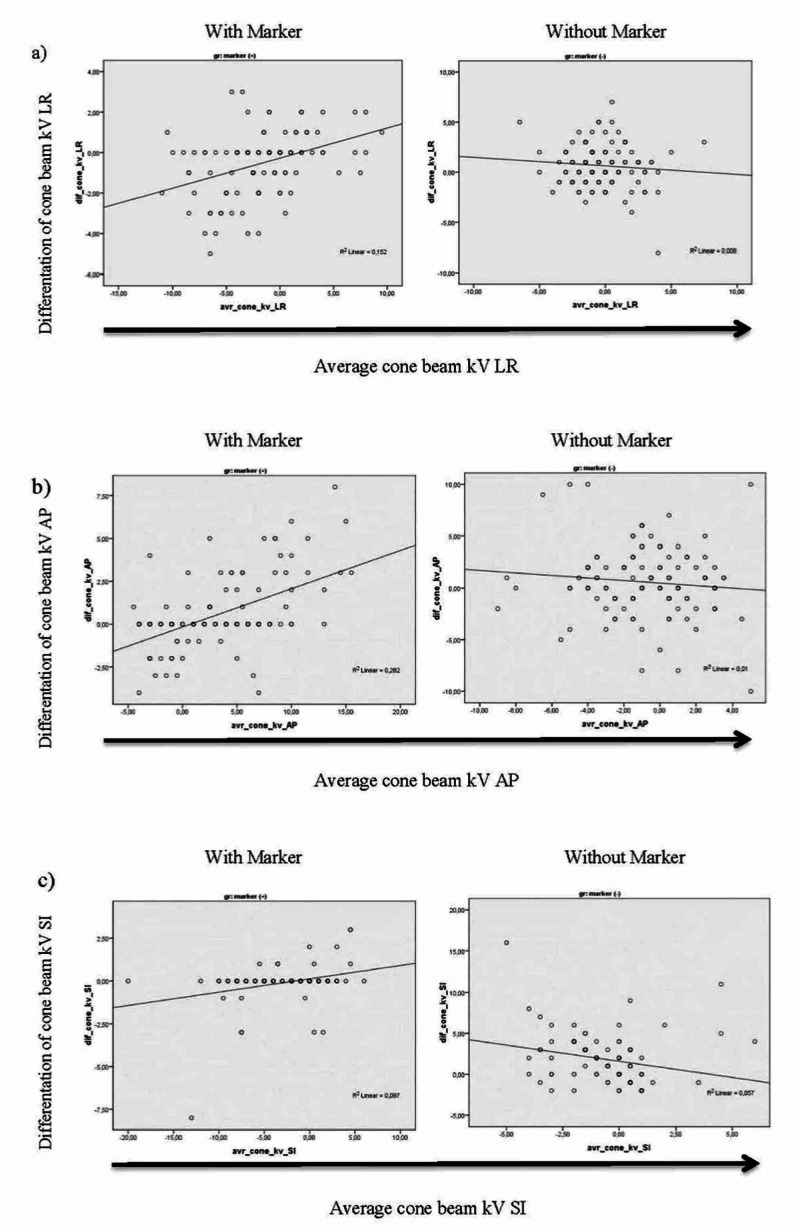
Bland-Altman error analysis Bland-Altman error analysis for cone-beam tomography (CBCT) vs kilovoltage (kV) portal image-based shifts with (left panel) and without (right panel) fiducial marker-based shifts for all 20 patients and all fractions in the LR (a), AP (b), and SI (c) directions.

Using the Bland-Altman analysis, R2 values for patients with markers in the LR, AP, and SI directions were 0.152, 0.282, and 0.097, respectively. R2 values for patients without markers in the LR, AP, and SI directions were 0.008, 0.01, and 0.057, respectively. The proximity of an R2 value to “0” means the existence of consistency between two sets of data compared by the Bland-Altman analysis. Our results indicate a larger difference between shifts comparing kV and CBCT imaging for all patients in both groups (with and without markers) in the LR direction (Figures 3a-3c). On the other hand, corrections for kV and CBCT, mean value, standard deviation, and 95% confidence limit (CL) values obtained for all patients with markers and without markers in the LR, AP, and SI directions and histograms belonging to these values are presented in Table 3.

**Table 3:** Estimates of group mean, standard deviation, standard error, and 95% confidence interval Estimates of group mean, standard deviation, standard error, and 95% confidence interval (min-max) values when comparing the two different methods in three directions: LR, AP, and SI (the results are based on 20 study patients)

			N	Mean	Std. deviation	Std. error	95% confidence interval	
							minimum	maximum
CBCT_AP		marker (-)	120	-,4500	3,06745	,28002	-1,0045	,1045
		marker (+)	120	3,5667	5,51352	,50331	2,5701	4,5633
		Total	240	1,5583	4,88580	,31538	,9371	2,1796
CBCT_SI		marker (-)	120	,3000	1,91237	,17457	-,0457	,6457
		marker (+)	120	-2,5333	4,42478	,40393	-3,3331	-1,7335
		Total	240	-1,1167	3,68574	,23791	-1,5853	-,6480
CBCT_LR		marker (-)	120	,0000	2,34072	,21368	-,4231	,4231
		marker (+)	120	-1,8917	4,28167	,39086	-2,6656	-1,1177
		Total	240	-,9458	3,57133	,23053	-1,4000	-,4917
kv_AP		marker (-)	120	-1,0333	3,35800	,30654	-1,6403	-,4263
		marker (+)	120	3,0000	4,43818	,40515	2,1978	3,8022
		Total	240	,9833	4,41656	,28509	,4217	1,5449
kv_SI		marker (-)	120	-1,5333	2,42859	,22170	-1,9723	-1,0943
		marker (+)	120	-2,4667	4,09741	,37404	-3,2073	-1,7260
		Total	240	-2,0000	3,39332	,21904	-2,4315	-1,5685
kv_LR		marker (-)	120	-,6583	2,50879	,22902	-1,1118	-,2048
		marker (+)	120	-1,3750	3,70569	,33828	-2,0448	-,7052
		Total	240	-1,0167	3,17807	,20514	-1,4208	-,6125

## Discussion

Early-stage prostate cancer is effectively treated with radiotherapy. Dose escalation has been shown to result in a better biochemical relapse-free survival in prostate cancer. While the survival rate of prostate cancer is high, late rectal and bladder complications due to high doses of radiotherapy remain an important morbidity. Maximum tumor control with less normal tissue toxicity can be provided with highly conformal radiotherapy. Not only do higher doses of IMRT improve the treatment results in terms of tumor control, but they also significantly decrease early- and late-term toxicities [7-10]. Quality control is crucial for both treatment planning and delivery, due to the often steep dose gradients and the complexity of the technique [11]. Furthermore, interfractional set-up errors and organ motion may cause treatment uncertainties, such as irradiating the normal tissue above the tolerance dose or the prostate below the planned doses, which both decrease the therapeutic ratio [12-13]. Two-dimensional (2D) or three-dimensional (3D) IGRT methods have been developed in order to enable more accurate patient setup and, subsequently, more accurate treatment delivery [14]. IGRT must be easy-to-use, easy-to-understand, user-independent, capable of rapid imaging and re-positioning, result in little additional radiation dose, and have adequate image quality for evaluation. Images should be reproducible and reliable for both planning and evaluation. However, as it stands today, there is no ideal IGRT method fulfilling all the aforementioned criteria.

This study compared online kV and CBCT imaging methods, which are two different IGRT techniques to perform target volume localization in prostate cancer patients immediately before treatment delivery. Similar studies exist in the literature. Moseley et al. compared MV and CBCT imaging for 15 patients with markers [15]. They reported that there was a relationship between MV and CBCT images for patients with markers, according to the Pearson correlation analysis. In that study, shifts performed within 3 mm tolerance for kV in the LR, AP, and SI directions were 99.6%, 70.3%, and 78%, respectively, whereas for CBCT, they were 87.4%, 41.3%, and 49.3%, respectively. The results of the study indicated that correlation in the LR dimension was superior to other dimensions because both the SI and AP dimensions are influenced by the difficulty in establishing the prostate-bladder junction as well as the prostate apex on CT imaging [15]. Our results are in accordance with Moseley et al.’s findings. In a similar study performed by Barney et al., 36 patients were evaluated using an online image assessment [16]. The authors observed consistency between kV and CBCT images for patients with markers, according to the Pearson correlation analysis. Comparing these results to those of our study, the correlation in the direction of LR for kV and CBCT is lower according to the Pearson correlation analysis. According to our results, shift values were within tolerance limits (3-5 mm) in all three dimensions. However, AP shifts appear to be the most affected by motion from adjacent organs such as the bladder and rectum. Moreover, after CBCT imaging, during the evaluation, the markers of patients were not removed. And this seems to be the most significant limitation of our study. This can be considered a bias. The study performed by Barney et al. also faced the same limitation [16]. Conversely, Moseley et al. overcame this limitation by using post-processing software, which removes markers after CBCT imaging for patients with markers [15].

The most important advantage of kV imaging when using markers is that the total treatment time, including localization and setup time, is less when compared to CBCT. This may be more important for clinics that have a higher throughput of patients. Further, CBCT imaging technology may not be readily available in every clinic, yielding orthogonal kV radiographs as the only option. However, CBCT has important advantages over kV: it provides much more information about the shape and location of the bladder and rectum in addition to the localization of the prostate [17].

Most prostate cancer patients are over 70 years of age and have additional comorbidity factors. For example, a proportion of cases are on continuous anticoagulant therapy and thus fiducial marker implantation may not be feasible. The surgical placement of fiducials is an invasive procedure, with potential complications, including a predisposition to resistant infections, bleeding, and displacement of the fiducials. These factors should be carefully considered before deciding to place fiducials in a given patient versus the alternative CBCT option. However, one disadvantage of CBCT is that prior to each treatment, a physician or an experienced technician is required to interpret the imaging and perform accurate matching in real-time. This further prolongs the treatment time. In contrast, the feasibility and familiarity of kV imaging performed by a technician make it a significantly easier and faster option for daily prostate IGRT.

## Conclusions

Although IGRT performed with implanted markers appears to provide more accurate targeting in terms of smaller deviation values, it may not be available in many instances, and, therefore, an alternative approach is needed. In this case, a kV-CBCT based IGRT approach without markers can be safely implemented and provide a reasonable alternative to the marker-based approach. An important advantage of CBCT is the avoidance of an invasive intervention, albeit at the expense of increased treatment delivery time and the presence of an experienced technician. The selected IGRT method should be tailored to the patient’s needs, taking into account multiple factors. Therefore, the decision to implant fiducials should be balanced against the relative contraindications, including comorbid conditions, the use of anticoagulation therapy, the potential risk for infectious and hemorrhagic complications of the procedure itself, and the risk of migration of the fiducial.
